# Influence of temperature and relative humidity on survival and fecundity of three tsetse strains

**DOI:** 10.1186/s13071-016-1805-x

**Published:** 2016-09-29

**Authors:** Soumaïla Pagabeleguem, Sophie Ravel, Ahmadou H. Dicko, Marc J. B. Vreysen, Andrew Parker, Peter Takac, Karine Huber, Issa Sidibé, Geoffrey Gimonneau, Jérémy Bouyer

**Affiliations:** 1 Pan-African Tsetse and Trypanosomosis Eradication Campaign, 01 BP 1087 Bobo-Dioulasso, Burkina Faso; 2 CIRAD, UMR15 CMAEE, F-34398 Montpellier, France; 3 IRD, UMR INTERTRYP, F-34398 Montpellier, France; 4 West African Science Service on Climate Change and Adapted Land Use, Climate Change Economics Research Program, Cheikh Anta Diop University, Dakar-Fann, Sénégal; 5 Insect Pest Control Laboratory, Joint FAO/IAEA Programme of Nuclear Techniques in Food and Agriculture, International Atomic Energy Agency, Vienna International Centre, PO Box 100, A-1400 Vienna, Austria; 6 Institute of Zoology, Section of Molecular and Applied Zoology, Slovak Academy of Sciences, Dúbravská cesta 9, 845 06 Bratislava, Slovakia; 7 INRA, UMR1309 CMAEE, F-34398 Montpellier, France; 8 Centre International de Recherche-développement sur l’Élevage en Zone Subhumide, BP 454 Bobo-Dioulasso, Burkina Faso; 9 CIRAD, UMR INTERTRYP, F-34398 Montpellier, France; 10 National Institute for Tsetse and Trypanosomosis Control and Eradication, Livestock Development Sector, Ministry of Agriculture, Addis Ababa, Ethiopia; 11 Pan-African Tsetse and Trypanosomosis Eradication Campaign coordination office, Rural Economy and Agriculture Department, African Union Commission, PO Box 3243, Addis Ababa, Ethiopia

**Keywords:** Tsetse flies, Area-wide integrated pest management, Sterile insect technique, Mass-rearing, Survival, Fecundity, Environmental conditions

## Abstract

**Background:**

Tsetse flies occur in much of sub-Saharan Africa where they are vectors of trypanosomes that cause human and animal African trypanosomosis. The sterile insect technique (SIT) is currently used to eliminate tsetse fly populations in an area-wide integrated pest management (AW-IPM) context in Senegal and Ethiopia. Three *Glossina palpalis gambiensis* strains [originating from Burkina Faso (BKF), Senegal (SEN) and an introgressed strain (SENbkf)] were established and are now available for use in future AW-IPM programmes against trypanosomes in West Africa. For each strain, knowledge of the environmental survival thresholds is essential to determine which of these strains is best suited to a particular environment or ecosystem, and can therefore be used effectively in SIT programmes.

**Methods:**

In this paper, we investigated the survival and fecundity of three *G. p. gambiensis* strains maintained under various conditions: 25 °C and 40, 50, 60, and 75 % relative humidity (rH), 30 °C and 60 % rH and 35 °C and 60 % rH.

**Results:**

The survival of the three strains was dependent on temperature only, and it was unaffected by changing humidity within the tested range. The BKF strain survived temperatures above its optimum better than the SEN strain. The SENbkf showed intermediate resistance to high temperatures. A temperature of about 32 °C was the limit for survival for all strains. A rH ranging from 40 to 76 % had no effect on fecundity at 25–26 °C.

**Conclusions:**

We discuss the implications of these results on tsetse SIT-based control programmes.

## Background

Tsetse flies are the cyclical vectors of human African trypanosomoses (HAT) and African animal trypanosomoses (AAT), which are debilitating diseases affecting humans (i.e. ‘sleeping sickness’) and livestock (i.e. ‘nagana’), respectively [1, 2]. The presence of tsetse flies impairs the development of sustainable and productive agricultural systems in over ten million km^2^ of sub-Saharan Africa [3, 4] leading to potential losses in livestock and crop production estimated at USD 4750 million annually [5]. In this context, vector control is considered an important component of the integrated management of both HAT [6] and AAT [1, 7–10].

*Glossina palpalis gambiensis* is one of the most important vectors of trypanosomes in West Africa [11, 12]. Over the past decades, chemotherapy-based management strategies of the disease has shown limitations linked to the development of parasite-resistance to the available trypanocidal drugs [13]. In addition, vector control programmes relying on the use of insecticides and that were not implemented following area-wide principles [14] failed to show sustainable results, with re-introduction of the flies from bordering infested areas as a consequence [15]. The use of the sterile insect technique (SIT) within an area-wide integrated pests management (AW-IPM) approach [16] appears necessary (at least in West Africa for this riverine species) to achieve eradication of this insect vector [17]. Recently, considerable efforts have been directed towards improving the application of this technique against *G. p. gambiensis* to support eradication programmes in Senegal and other countries [18–24]. Indeed, previous work carried out in Mali and Senegal showed that the Burkina Faso (BKF) strain of *G. p. gambiensis* competes with *G. p. gambiensis* populations from other countries [19, 25, 26], thus indicating the potential of the BKF strain to be used in eradication strategies of isolated populations of this species in selected areas of West Africa such as the Niayes in Senegal [10, 27].

Little is known, however, of the relationship between tsetse flies and environmental factors such as temperature and relative humidity (rH) [28, 29]. Previous research in this area has focused on the pupa stage and, specifically, on impacts of environmental variability on pupal water loss [30, 31], the effect of dehydration on mortality and adult emergence [30] and metabolic responses [32]. Previous studies on adult tsetse flies were conducted mostly on the *morsitans* group flies, and they focused on temperature-dependent metabolic rate variation [33–36] and thermic tolerance [34, 37].

In nature, climatic parameters influence the spatial and temporal distribution, abundance and behavior of insects [17, 34, 38–41]. Although the BKF strain is compatible with strains from other countries [19], seasonal variations within one country and between countries could affect important components of fly competitiveness, i.e. mating performance and survival. High temperatures and low humidity are detrimental for the survival of tsetse flies [28, 34, 37] and under such circumstances flies will seek resting places with more favourable microclimatic conditions, i.e. higher humidity and lower temperature [42].

In view of results obtained in field cage studies and during pilot trial releases, it was decided to use the BKF strain for release in the Senegal project. In spite of data that indicated adequate compatibility and competitiveness of the BKF strain with the local Senegal populations, two new strains were developed to serve as alternatives in case of failure of the BKF strain to perform in certain ecosystems of the Niayes. These two strains were a *G. p. gambiensis* Senegal (SEN) strain that originated from Pout/Sebikotane in the Niayes and an introgressed (SENbkf) strain, obtained from crossing BKF females with SEN males. Whilst the BKF strain had been cultured for more than four decades, and was more prolific than the newly created SEN strain [19], it was hypothesized that a strain introgressed with the BKF strain, adapted to an artificial mass-rearing environment but maintaining most of the SEN genetic background, may lead to better adaptation to the harsh environment of the Niayes and, therefore, better performance in certain ecosystems when compared to the BKF strain. In the Niayes area of Senegal, the ecology of *G. p. gambiensis* populations from which SEN was obtained is different from that of other *G. p. gambiensis* populations that thrive in riparian forests. In the Niayes, the habitat favoured by *G. p. gambiensis* includes mainly mango and citrus tree plantations, residual riparian thickets and palm tree plantations, as the flies have adapted to this man-made vegetation and strong anthropic pressures [8, 17, 43]. Moreover, the combined use of markers such as microsatellites and mitochondrial DNA and wing morphometrics showed that the Niayes population was completely isolated from the main tsetse belt in West Africa [10, 27] and can thus be considered a different ecotype or even sub-species [44].

While best environmental conditions for rearing the BKF strain are 24–25 °C, 75 ± 5 % rH and 12 h:12 hrs light (L): dark (D) regime [45–47], the optimal conditions for the SEN and SENbkf strains remain unknown. In view of the differences in environmental conditions between regions, it is therefore crucial to determine which strain would perform best in which particular ecosystem.

## Methods

### Fly strains

Three strains of *G. p. gambiensis* were used in this study: BKF, SEN and SENbkf. The fly material of the BKF and SEN strains was derived from colonies maintained at the Insect Pest Control Laboratory (IPCL) of the Joint FAO/IAEA Programme of Nuclear Techniques in Food and Agriculture, Seibersdorf, Austria and the SENbkf flies were derived from a colony developed at the IPCL and maintained at the Slovak Academy of Sciences (SAS), Bratislava, Slovakia.

The BKF strain was established at Maisons-Alfort, France in 1972 using material collected in Guinguette, near Bobo-Dioulasso, Burkina Faso. It was transferred in 1975 to the Centre de Recherche sur les Trypanosomiases Animales (CRTA), Burkina Faso [45, 47] [CRTA was later renamed Centre International de Recherche-Développement sur l’Elevage en zone Subhumide (CIRDES)]. In 2009, 8000 pupae of this colony were shipped to the IPCL to establish a colony for research purposes to support the eradication programme in the Niayes [19, 21]. The IPCL colony provided seed material to the SAS where a colony was likewise established to supply additional pupae to the Senegal project.

The SEN strain was established at the IPCL from September 2009 to December 2010 from pupae obtained from wild females collected in Pout and Sebikotane and that were shipped weekly to the IPCL [19].

The SENbkf strain was developed in 2010 at the IPCL and then transferred to the SAS insectary. Initial crosses were made between SEN males and BKF females and the hybrid females were backcrossed 4 times with SEN males. Flies of the 5th generation were then intercrossed for rearing, initially at the IPCL and later at the SAS. The strain is, therefore, genetically composed of 97 % of the SEN genome and 3 % of the BKF genome, with mitochondria from the BKF strain.

These colonies were maintained in both insectaries at 24–25 °C and 75 ± 5 % rH with a 12 L:12D cycle. The flies were offered blood meals using an in vitro silicon membrane feeding system using bovine blood (Svaman SRA, Majava, Slovak Republic), frozen at -20 °C and irradiated with 1 kGy in a commercial irradiator [48].

### Preliminary data from source insectaries

The SENbkf and SEN strains were reared at the SAS and IPCL, respectively, while the BKF strain was reared in both institutes. Production data of the SENbkf and SEN strains were collected from August 2012 to October 2014 and from January 2010 to September 2014, respectively. Production data of the BKF strain were collected from January 2011 to September 2014 at the IPCL and from April 2010 to September 2014 at the SAS. The weekly datasets of the 3 colonies were analyzed with colony size, daily mortality and fecundity being the main parameters.

### Transport of pupae

Pupae of the SENbkf strain were transported from the SAS to the IPCL, where pupae of the BKF and SEN colonies were added and shipped with a courier service to the Centre de Coopération Internationale en Recherche Agronomique pour le Développement (CIRAD), Montpellier, France. Pupae were placed in Petri dishes with the top lid perforated with holes of ~ 2 mm diameter for aeration. The Petri dishes were placed in a kraft paper air bubble envelope (TAP Comebag® type B (11 × 21.5 cm)) to absorb mechanical shocks during transport. Due to the small size of the SENbkf colony, pupae were collected over 1 week and pooled to constitute 1 batch while for BKF and SEN strains, pupae of the same batch had the same age. There were 2 batches for each shipment and for each strain. On the day of shipment, pupae of batch 1 were 15 days old for the BKF and SEN strains and 9–15 days old for the SENbkf strain, and those of batch 2 were 8 and 2–8 days old, respectively. Each batch of the BKF/SEN and the SENbkf strains contained on average 200 and 50 pupae, respectively. Pupae were shipped on Tuesdays and were received on Fridays.

### Experimental conditions

Pupae were divided on receipt into glass Borel jars (Dutscher Scientific, Essex, UK) of 3.5 cm diameter and 8.2 cm in height (40 pupae per tube) and kept in an incubation room (25 ± 1 °C, 75 ± 5 % rH, 12 L:12D) until emergence. Every morning (except Saturday and Sunday), the emerged flies were transferred to Roubaud cages and placed in climate controlled rooms with adjustable temperature, rH and photoperiod. A climate chamber (Memmert HPP 110, GmbH & Co KG, Schwabach, Germany) with an internal size 40 × 25 × 32 cm was also used for the 26 °C, 40 % rH treatment. A data logger (KistockKTH-350A, KIMO, Montpon, France) was placed inside each room and was programmed to display temperature and relative humidity every minute and to record data every 5 min. The data loggers have a resolution of 0.1 °C and 0.1 % rH and an accuracy of ± 0.3 °C and ± 1.5 % rH.

The maximum critical temperature was evaluated at temperatures ranging from 25 °C to 35 °C with an increment of 5 °C and the rH fixed at 60 %. The minimum critical rH was assessed at a constant temperature of 25 °C and a rH of 40, 50, 60 and 75 %. Flies belonging to the same cohorts were used to assess the survival and fecundity. The fecundity was assessed in all treatments where the longevity of flies allowed it. A photoperiod of 12 L:12D was maintained for all experimental rooms and light intensity varied from 280 to 500 Lux depending on the position of the cages in the room.

### Fly handling

Newly emerged flies were separated by sex and put into Roubaud cages (maximum 25 flies per small cage (7.5 × 5 × 4 cm) and 40 flies per large cage (13.5 × 8 × 4.5 cm) and then placed in the experimental rooms. Due to the low number of flies that emerged per strain and sex on some days, the number per cage was often less than the maximum (≤ 40 flies) and each constituted a replicate. Females were put into cages covered with white tulle of large mesh (2.5 mm) and males in smaller mesh cages (1.5 mm). The large mesh allowed third instar larvae (L3) to escape from the cage. Flies were offered a blood meal three times a week (Monday, Wednesday and Friday) on an in vitro silicon membrane system using defibrinated sheep blood collected aseptically and previously frozen at -20 °C. The feeding system was installed in a climatic room that was maintained at 25 ± 1 °C and 50 ± 5 % and the system was used for feeding flies from all treatments. The flies remained in the feeding room for less than 30 min.

Three to 4 day-old virgin females were mated with 6–8 day-old virgin males (the time that the flies become sexually mature) [49] and put into holding cages at an initial male to female ratio of 1:3. Males and females remained together until all flies had died. Due to the low number of females on some days, the mating was often done in small number and each constituted a replicate.

### Fecundity and reproductive biology

Mating cages were placed in individual larviposition cups and pupae were collected daily (except Saturday and Sunday) and sorted into normal and aborted L3. The normal pupae were weighed using an electronic balance of 0.1 mg sensitivity and automatic calibration (Precisa® 410 AM-FR, HE electronic, Kadikoy Istanbul, Turkey). The production of pupae was recorded daily by treatment and cage. The first larval period (time between female emergence and the production of the first pupae) and the subsequent interlarval period (time between the reproductive cycles) was also recorded.

### Mortality

Mortality was recorded daily (except on Saturdays and Sundays) for each treatment per strain and per sex until the death of the last individual. Dead flies were sorted into blood-fed and starved fly mortalities.

### Adult emergence rate

Pupae were kept in an incubation room at 25 ± 1 °C and 75 ± 5 % rH. The number of flies that emerged per treatment was recorded daily (except on Saturdays and Sundays) and used to calculate the percentage of emerged adults from the total number of pupae for each treatment. Only flies that escaped from the pupal case were considered as emerged.

### Data analysis

The R Software (version 3.1.0) was used to perform all statistical analyses [50]. The survival of flies kept under various experimental conditions was analyzed using Kaplan-Meier survival curves. Survival curves were compared using the coxph model [51] where the strain, the sex, the temperature, rH and their second and third order interactions were used as explanatory variables and survival rate as the response variable. The best model was selected on the basis of the lowest corrected Akaike information criterion (AICc), and the significance of the fixed effect was tested using the likelihood ratio test [52, 53]. When analyzing mortality, we considered the mean temperature and humidity to which the flies were subjected the day before their death, to account for potential variability of the conditions within the climatic rooms. For the fecundity analysis, we considered their mean values over 10 days before each larviposition. Pairwise comparisons of median survivals between treatments were tested with a Tukey’s *post-hoc* test (‘*glht*’ function in the ‘*multcomp*’ package). The best model was used to plot the survival rate against the maximum temperatures from 24 to 36 °C. The optimal temperature for rearing the BKF strain is 25 ± 1 °C [45–47]. Thus, from the plot for females, the mean survival corresponding to the maximum temperature for BKF mass-rearing (the reference strain), i.e. 26 °C, was used to determine the maximum temperature for the mass-rearing of SEN and SENbkf strains.

The pupal production was followed by cage and not individually, thus the first larval period was determined per cage. The number of pupae per cage was plotted against the age of females and the first peak was considered as corresponding to the first larval period. The subsequent interlarval interval was determined by considering the following peak. When there were 2 peaks within less than 7 days, the highest peak was used. The first and subsequent interlarval periods, the pupal production and the pupal mass were analyzed using general linear models [54] where the strain, the treatment and their interactions were considered as fixed variables. For modeling the first and subsequent interlarval periods, the cage number was considered as a random effect while for modeling the pupal mass, the random effect was the emergence date. The smallest AICc of different models was used to choose the best model and the significance of the fixed effect was tested using the likelihood ratio test.

## Results

### Experimental conditions

The data recorded with the Kimo® loggers showed that during the experiments, temperature and rH varied around the expected values. Table 1 gives the target values and the measured mean temperatures and rH experienced by the flies. In subsequent analyses, we therefore used the mean recorded conditions instead of the target values.

**Table 1:** Target temperature and relative humidity and mean (± standard deviation) environmental conditions experienced by the flies and recorded with the Kimo® loggers

Target conditions (°C–% rH)	Recorded conditions	
	Temperature (°C)	Relative humidity (%)
Experimental rooms		
25–50	25.2 ± 0.5	47.7 ± 8.5
25–60	26.4 ± 0.2	55.2 ± 1.6
25–75	25.1 ± 0.4	76.1 ± 8.0
30–60	31.4 ± 0.8	55.1 ± 2.0
35–60	35.3 ± 1.3	50.2 ± 2.5
Experimental chamber^a^		
26–40	26.0 ± 0.0	40.3 ± 0.7
Pupal incubation room		
25–75	25.6 ± 0.3	73.4 ± 3.3

^a^ *Memmert HPP 110*

A total of 5984 pupae were received from the ICPL (BKF and SEN) and SAS (SENbkf) insectaries, of which 2883, 2245 and 856 were of the BKF (5 shipments), SEN (6 shipments) and SENbkf (7 shipments) strains, respectively. The emergence rates of the adult flies in the pupal incubation room (25.6 °C, 73.4 % rH) were 95.1 %, 87.3 % and 84.4 % for the BKF, SEN and SENbkf strains, respectively. Table 2 shows the number of flies by strain, by gender and by treatment used in the experiments. Almost all females that survived until the mating date were used for fecundity measurements.

**Table 2:** Number of flies used for the experiments per strain, sex and treatment

Treatment (°C–% rH)	BKF		SEN		SENbkf	
	♂	♀	♂	♀	♂	♀
25.2 ± 0.5–47.7 ± 8.5	270	465	173	310	40	87
26.4 ± 0.2–55.2 ± 1.6	141	219	145	219	82	48
25.1 ± 0.4–76.1 ± 8.0	169	150	188	122	90	97
26.0 ± 0.0–40.3 ± 0.7	85	172	112	130	51	52
31.4 ± 0.8–55.1 ± 2.0	273	179	99	56	16	17
35.3 ± 1.3–50.2 ± 2.5	217	134	86	61	12	20
Total	1155	1319	803	898	291	321

### Production parameters of the 3 colonies in the insectaries of origin

**Fig. 1:**
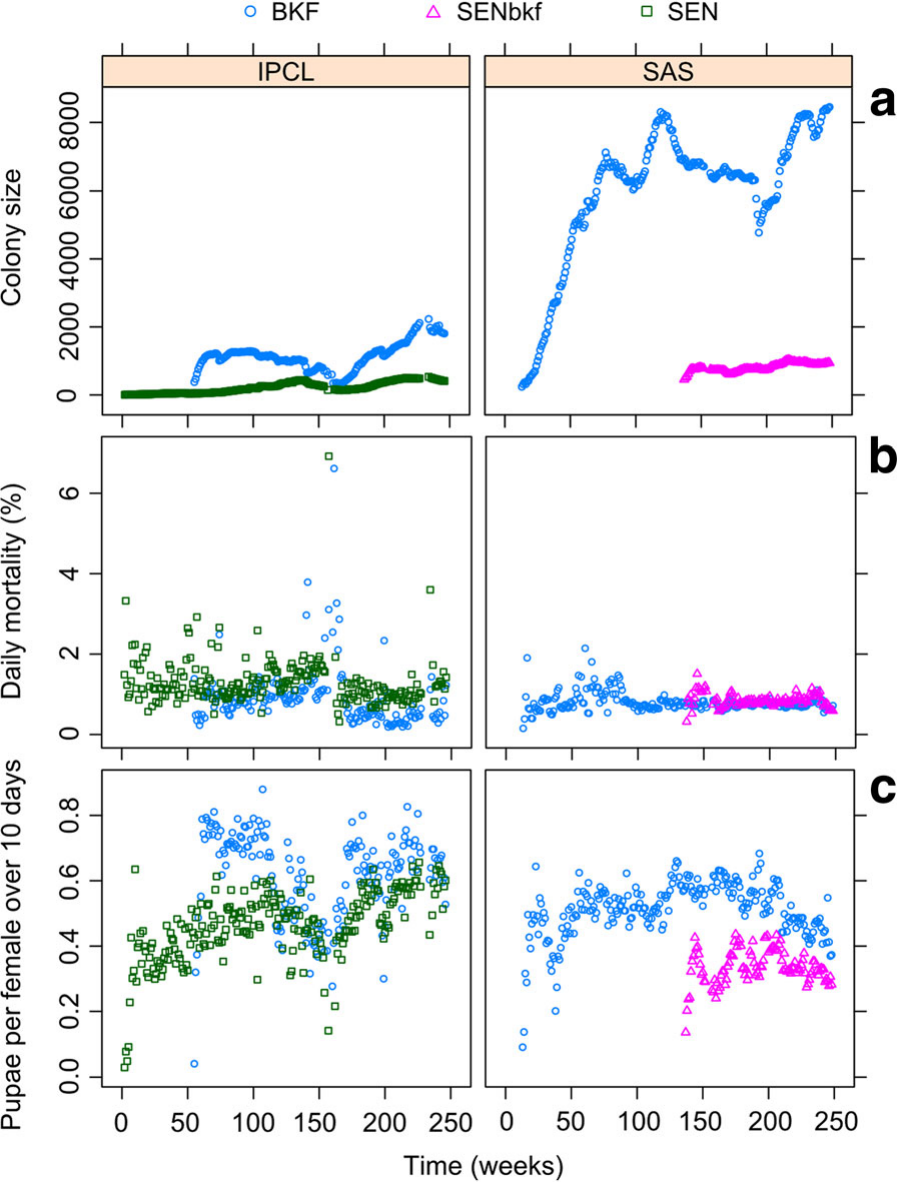
Performance parameters of the *Glossina palpalis gambiensis* strains (BKF, SEN and SENbkf) in the insectaries of origin (IPCL and SAS). The time was recoded in weeks from 2010. **a** Temporal fluctuations in colony size (females), **b** daily mortality (%), and **c** fecundity

Figure 1a shows the temporal fluctuations in colony size (females) of the 3 strains in the insectaries of origin. At the IPCL, the daily mortality (mean ± SD) was significantly higher (*P* < 10^-3^; Table 3) for the SEN flies (1.3 ± 0.5 %) than that of the BKF strain (0.9 ± 0.6 %) while in the SAS insectary, the mortality was similar between SENbkf (1.0 ± 0.2 %) and BKF (1.0 ± 0.3 %) flies (Fig. 1b). Considering the 3 strains, the BKF flies had the lowest mortality, followed by the SENbkf, and the SEN flies (*P* < 10^-3^; Table 3). The fecundity was better for the BKF than the SEN and the SENbkf flies, but the SEN colony performed better than the SENbkf colony (*P* < 10^-3^; Table 3; Fig. 1c). More importantly, fecundity of the SEN colony increased significantly over time (*P* < 10^-3^; Table 3) but not that of the SENbkf colony. When restricting the analysis to the BKF colonies reared in the 2 insectaries, a lower mortality and a better fecundity was observed for the colony maintained at the IPCL insectary when compared with the SAS colony (*P* < 10^-3^; Table 3).

**Table 3:** Summary of the best mixed effect model results for the preliminary data from source insectaries and the experimental fecundity

Trait	Fixed effect	Estimate	Standard Error	*Z-*value	P(> |z|)
Preliminary data from source insectaries					
Mortality	(Intercept)	-2.795867	0.016912	-165.32	<2e-16***
	SAS insectary	-0.021391	0.003165	-6.76	1.4e-11***
	SENbkf	0.143523	0.004638	30.95	<2e-16***
	SEN	0.529115	0.005628	94.02	<2e-16***
Production	(Intercept)	-0.212193	0.012092	-17.55	<2e-16***
	SAS insectary	-0.320035	0.001496	-213.88	1.4e-11***
	SENbkf	-0.633439	0.00255	-248.40	<2e-16***
	SEN	-0.382742	0.00319	-119.99	<2e-16***
Experimental fecundity					
First larval period	(Intercept)	20.5769	0.3254	63.244	<2e-16***
	BKF	-0.6436	0.5379	-1.196	0.23614
	SEN	-1.4465	0.4749	-3.046	0.00342**
Interlarval period	(Intercept)	8.4706	0.5991	14.139	<2e-16***
	SENbkf	1.9294	0.9844	1.96	0.0572.
	SEN	1.8627	0.875	2.129	0.0396*
Pupae per initial female	(Intercept)	3.1248	0.2986	10.464	<2e-16***
	SENbkf	-0.9939	0.3986	-2.494	0.0148*
	SEN	-0.5463	0.4314	-1.266	0.2092
Pupal mass	(Intercept)	20.6968	0.3743	55.296	<2e-16***
	BKF	0.7434	0.393	1.891	0.0631.
	SENbkf	-0.6608	0.3431	-1.926	0.0585.
	26.4 °C–55.2 %	-2.1292	0.396	-5.377	1.14e-06***
	25.2 °C–47.7 %	-0.2925	0.3994	-0.732	0.4666
	26.0 °C–40.3 %	-0.6103	0.4579	-1.333	0.1873
Adult emergence	(Intercept)	2.9945	0.1545	19.387	<2e-16***
	SENbkf	-1.0086	0.2229	-4.524	6.07e-06***
	SEN	-0.9223	0.155	-5.95	2.68e-09***
	26.4 °C–55.2 %	-0.486	0.2303	-2.11	0.0349*
	25.2 °C–47.7 %	0.4255	0.2601	1.636	0.1018
	26.0 °C–40.3 %	0.3428	0.2192	1.563	0.118

Significance: ***P ≤ 0.001; ** *P* ≤ 0.01; *P ≤ 0.05 (these apply to values above)

### Relationship between survival and environmental conditions

**Fig. 2:**
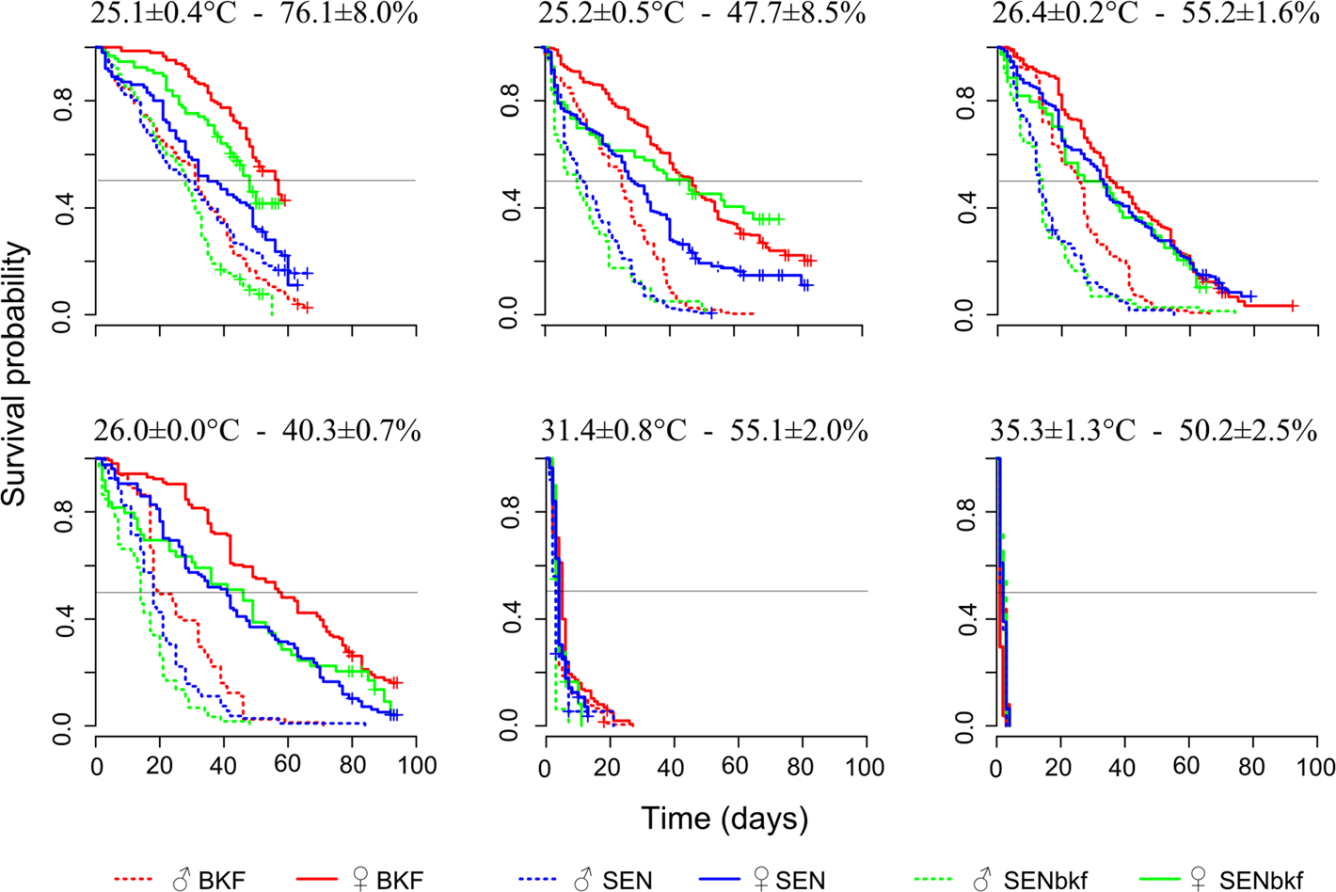
Survival curves of flies by treatment, strain and sex

**Fig. 3:**
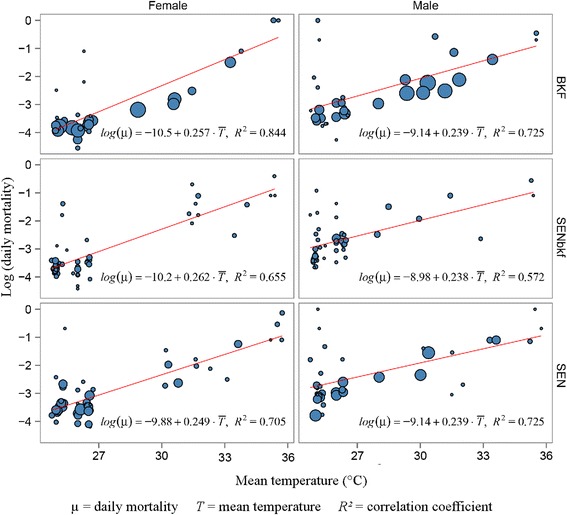
Correlation between daily mortality and mean temperature for male and female flies of the BKF, SEN, and SENbkf strains. The size of the data points is proportional to the number of flies at each date of emergence with the equation of the linear model and *R* ^*2*^ also supplied

Survival curves of the flies from the different treatments are presented by strain and gender in Fig. 2. The median survival times obtained from the curves are summarized in Table 4. The analyses showed that survival of flies was influenced by strain, sex and temperature (*P* < 10^-3^; Table 5). Females survived significantly longer than males, irrespective of the strain and treatment (*P* < 10^-3^; Table 5). Survival was very short at high temperatures, i.e. at 31.4 °C the median survival was 4 days for females and 3 days for males of all strains, while at 35.3 °C the median survival was 2 days for both sexes (Table 4). Within the same gender, at 25–26 °C there was little difference in survival between 40 and 76 % rH (Fig. 2, Table 4). Under these environmental conditions, the median survival for females was 46.7, 42.5 and 37 days for BKF, SENbkf and SEN flies, respectively. For males, it was 27.3, 16.8 and 18.5 days respectively. These results show that the rH (40–76 %) has a marginal effect on survival (*P* = 0.06; Table 5). Overall, BKF flies survived longer than SENbkf and SEN flies irrespective of gender, and SENbkf female flies survived longer than SEN females but not males (*P* < 10^-3^; Table 5). The relationship between daily mortality and mean temperature for the 3 strains is presented by gender in Fig. 3.

**Table 4:** Median survival (days) by sex, strain and treatment

Treatment (°C–% rH)	Female			Male		
	BKF	SEN	SENbkf	BKF	SEN	SENbkf
25.1 ± 0.4–76.1 ± 8.0	57^a^	37^bc^	48^ab^	32^c^	30^c^	28^ce^
25.2 ± 0.5–47.7 ± 8.5	47^ab^	29^c^	46^ab^	25^ce^	13^d^	11^d^
26.4 ± 0.2–55.2 ± 1.6	36^bc^	33^bc^	30^bc^	25^ce^	13^d^	14^df^
26.0 ± 0.0–40.3 ± 0.7	58^a^	41^bc^	46^ab^	20^ef^	18^def^	14^df^
31.4 ± 0.8–55.1 ± 2.0	5^g^	4^gh^	4^gh^	3^hi^	3^hi^	3^hi^
35.3 ± 1.3–50.2 ± 2.5	1^j^	2^k^	3^ik^	2^k^	2^k^	2^k^

Median survivals were separated using Tukey’s *post-hoc* test and values that have a common letter (a-k, amongst columns and rows) are not significantly different (*P* > 0.05)

**Table 5:** Summary of the best cox model for the survival of flies

Fixed effect	Coef	Exp(coef)	SE(coef)	*Z*–value	P(> |z|)
Relative humidity	-0.002520	0.997483	0.001334	-1.889	0.05892.
Temperature	0.531286	1.701118	0.010805	49.172	<2e-16***
SENbkf	2.183868	8.880592	0.671772	3.251	0.00115**
SEN	3.173181	23.88334	0.439600	7.218	5.26e-13***
Male	5.949196	383.4449	0.344772	17.255	<2e-16***
Temperature × SENbkf	-0.076943	0.925942	0.024771	-3.106	0.00190**
Temperature × SEN	-0.105743	0.899656	0.016044	-6.591	4.37e-11***
Temperature × Male	-0.185816	0.830426	0.012024	-15.45	<2e-16***
SENbkf × Male	-1.327588	0.265116	0.900588	-1.474	0.14045
SEN × Male	-2.860653	0.057231	0.566269	-5.052	4.38e-07***
Temperature × SEN × Male	0.058313	1.060046	0.033291	1.752	0.07985
Temperature × SENbkf × Male	0.100787	1.106041	0.020437	4.932	8.16e-07***

*Abbreviation*: *Coef* coefficient, *SE* standard error

Significance: ***P ≤ 0.001; ** *P* ≤ 0.01 (these apply to values above)

**Fig. 4:**
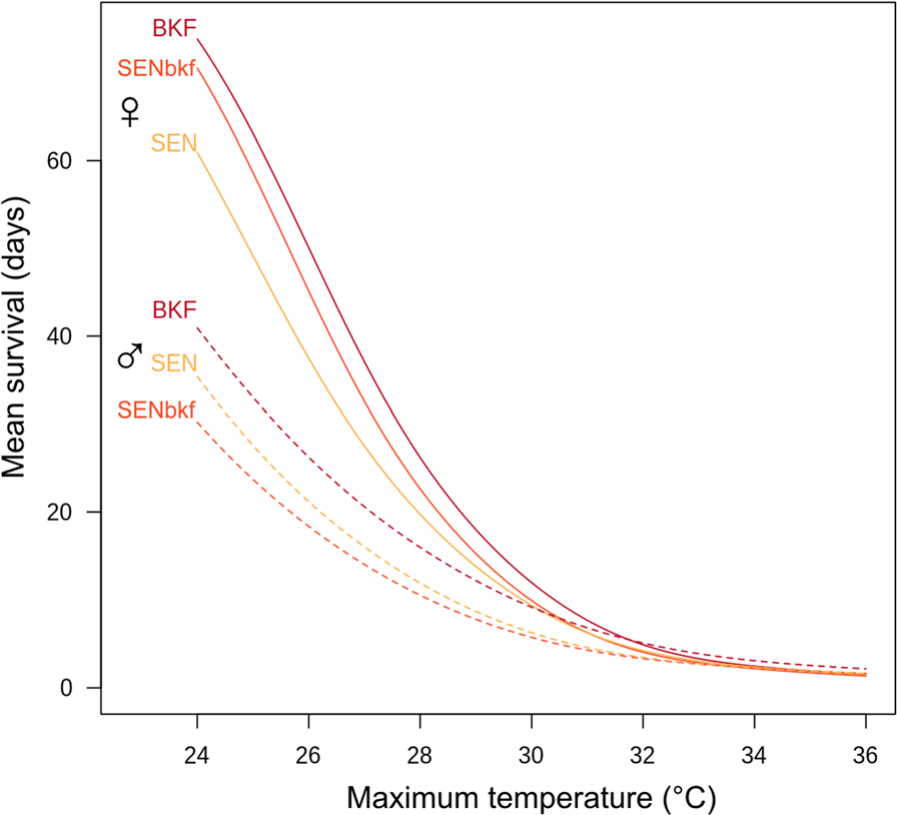
Mean survival of the BKF, SEN, and SENbkf strains plotted against the maximum temperature. The relative humidity was maintained constant at 60 %

The mean survival for the 3 strains (males and females) against the maximum temperature, i.e. the range of 24–36 °C (at 60 % rH) using the binomial mixed model showed a decrease in survival with increasing temperature (Fig. 4). There were negative effects on the interactions between temperature and the SEN and SENbkf strains on survival (*P* < 10^-3^; Table 5), showing that BKF flies resisted better at higher temperatures than SENbkf and SEN flies irrespective of gender. Females of the SENbkf strain had a similar mean survival than BKF females at moderate temperatures, whereas at higher temperatures, the resistance of SENbkf females decreased faster than that of BKF females until they reached the same level as SEN females (Fig. 4). Males of the SENbkf strain had the lowest resistance to increasing temperatures when compared with the BKF and SEN males (Fig. 4). The introgression thus showed increased resistance to high temperatures for females but not for males. Above 32 °C, all flies, irrespective of the strain and gender, died rapidly.

From the plot, the mean survival for BKF females at 26 °C (maximum temperature for BKF mass rearing) was 50 days (Fig. 4). This survival value corresponded to a maximum temperature of 25.6 and 24.9 °C for the SENbkf and SEN females, respectively.

### Fecundity in relation to environmental conditions

Fecundity of the different strains was assessed using a range of rH values (40–76 %) and a temperature of 25 and 26 °C, since above 30 °C survival was too low to monitor fecundity.

At 25–26 °C and between 40 and 76 % rH, the first larva of the SENbkf strain was deposited on average on day 19.7, which was similar to that of the BKF strain (day 19.2) (*P* = 0.2; Table 3), but significantly later than that of the SEN strain (day 19.0) (*P* = 0.003; Tables 3 and 6). These results indicate that the rH had no influence on the first larviposition day (*F*_(3,64)_ = 1.96, *P* = 0.1).

**Table 6:** First larviposition day, interlarval period, fecundity, mean pupal mass and emergence rate of adults under various experimental conditions

Treatment (°C–%)	First larviposition day			Interlarval period (days)			PPIF			Mean mass of pupae (mg ± sd)			Emergence (%)		
	BKF	SEN	SENbkf	BKF	SEN	SENbkf	BKF	SEN	SENbkf	BKF	SEN	SENbkf	BKF	SEN	SENbkf
25.1 ± 0.4–76.1 ± 8.0	21	20	19	9.3	8.0	10.0	3.2^ab^	2.7^ab^	2.1^ab^	21.6 ± 1.5^a^	21.0 ± 2.3^a^	19.4 ± 2.7^ab^	93.4	87.7	93.5
26.4 ± 0.2–55.2 ± 1.6	19	17	20	9.4	10.0	12.0	3.7^ab^	3.3^ab^	1.9^ab^	18.4 ± 2.8^b^	17.9 ± 3.2^b^	18.6 ± 1.8^b^	88.1	80.7	77.1
25.2 ± 0.5–47.7 ± 8.5	19	20	19	7.3	9.8	11.0	2.7^a^	1.8^b^	1.8^b^	21.3 ± 2.2^a^	20.7 ± 2.4^a^	18.9 ± 2.7^b^	94.9	89.5	89.0
26.0 ± 0.0–40.3 ± 0.7	20	19	19	8.0	11.6	9.3	3.4^ab^	2.5^ab^	2.7^ab^	20.7 ± 2.5^a^	20.4 ± 3.5^a^	19.3 ± 2.4^ab^	95.7	91.7	92.0

Abbreviation: *PPIF* pupae production per initial female at 56 days (8 weeks)

For the PPIF and mean mass of pupae, the values that have a common letter (a and ﻿b, amongst columns and rows) are not significantly different (*P* > 0.05)

For all strains, at 25–26 °C the rH also had no effect on the interlarval period (*F*_(3,42)_ = 0.35, *P* = 0.8). Overall, the analysis showed a significantly shorter interlarval period for BKF (8.5 days) females as compared with SEN (10.3 days, *P* = 0.04; Table 3) and SENbkf (10.4 days, *P* = 0.06; Table 3) females.

Results indicate that pupal production was significantly influenced by the *Glossina* strain (*F*_(2,80)_ = 3.19, *P* = 0.04) but not by the treatment (25–26 °C and 40–76 % rH) (*F*_(3,80)_ = 1.68, *P* = 0.2). Indeed, the highest mean pupae per initial female (PPIF) at 56 days (8 weeks), 3.2 (all treatments together except 31.4 and 35.3 °C treatments) recorded for the BKF strain (Table 6), was significantly higher than that for the SENbkf strain (2.1; *P* = 0.01; Table 3) and similar to that for the SEN strain (2.6; *P* = 0.2; Table 3). The strain effect was marginal as it was only observed at 25.2 ± 0.5 °C–47.7 ± 8.5 % rH.

At 25–26 °C, the mean pupal mass was relatively similar between strains, irrespective of the experimental rH (*P* = 0.06; Table 3 and 6); mean (± SD) values of 20.6 ± 1.4, 19.8 ± 1.7 and 19.4 ± 1.3 mg were obtained for the BKF, SEN and SENbkf strain, respectively. On the other hand, adult emergence was significantly better for BKF flies (93.5 ± 9.4 %) as compared with SEN (87.6 ± 18.5 %) and SENbkf (87.9 ± 19.0 %) flies (*P* < 10^-3^; Table 3). There was a marginal effect of rH on pupal mass and adult emergence as the lowest values were observed at 26.4 ± 0.2 °C and 55.2 ± 1.6 % rH (*P* < 10^-3^; Tables 3 and 6).

## Discussion

The aim of this study was to assess the effect of different temperatures at the same rH, but also to assess the effect of variation in rH at a single optimal temperature to try to determine the best environmental conditions for mass-production of the new *G. p. gambiensis* strains (SEN and SENbkf) and to define the critical maximum temperature and critical minimum rH for each strain. These aspects are important because, in 2000, the African Heads of States and Governments decided to increase efforts to address the tsetse and trypanosomosis problem on the African continent under the auspices of the African Union (PATTEC) [55]. For West Africa, this entails AW-IPM programmes against *G. p. gambiensis*; this species thrives in riparian and forest environments, and eradication strategies will require the use of the SIT. The results of this study will facilitate optimization of the mass-rearing of the three strains that are currently established and guide programmes that include a SIT component to select the strain that is best adapted to the local environmental conditions of the target area.

We found that female *G. p. gambiensis* survived significantly longer than males irrespective of the strain and environmental conditions except at sub-lethal temperatures. The difference in lifespan between the sexes is common in insects and it seems to be genetically determined [56]. Indeed, similar results were obtained with *Anopheles arabiensis* and *An. funestus* under combinations of temperature and humidity [57], with *Aedes albopictus* at constant temperatures [58, 59], and with *Ae. krombeini* at both constant and fluctuating temperatures [60]. In mosquitoes, this gender-biased difference seems to be associated with variations in the amount and composition of cuticle lipids between the sexes [61] which influences water loss [62], but also to differences in size [61]. Indeed, the larger mass of females translates into a higher water content, which further contributes to enhanced survival time [61]. These same factors could be implicated in the difference in survival of tsetse, as confirmed by pronounced differences in size between males and females.

At higher temperatures, survival of the flies was very low, i.e. the median survival was four days at 31 °C and 2 days at 35 °C for the two sexes (at 60 % rH). Similar results were obtained with *G. fuscipes fuscipes* at 30 °C and 19 % rH [63]. Mellanby [63]observed that at a temperature of 30 °C, the flies died quicker at a higher than at a lower humidity. *G. f. fuscipes* [63], *G. morsitans* [64], *G. tachinoides* and *G. m. submorsitans* [49] can survive at sub-lethal temperatures by evaporating water, but they can only do so for a short period (a few hours) and when the rH is low. In wild-caught *G. pallidipes*, the median survival at 37.9, 36.2 and 35.6 °C (95 % CIs: ± 0.5 °C) were 1, 2 and 3 h, respectively [34].

The BKF strain showed better survival when compared with the SEN and SENbkf strain even at high temperatures irrespective of gender. Such differences have been reported among species and determine their ability to survive in some environments [65]. For example, teneral *G. f. fuscipes* and *G. morsitans* exposed for 24 h to 25 °C and 50 % rH lost 3.2 and 2.7 mg water, respectively, for a mean mass of 22 and 19 mg [65]. This difference seems related to size; however, this appeared not to be the case with our *G. p. gambiensis* strains that had the same size. Our results suggest that this capability to survive at higher temperatures might also vary among populations of the same species. This observation supports earlier findings that the water balance response to variation in temperature and rH in *Glossina* varies within and among species, subgroups, and ecotypes, in terms of both magnitude of effects and direction of change [29]. The variation between populations of the same species in the ability to survive to higher temperatures is supported by the differences in habitat preferences observed between these two populations [8, 43, 66, 67]; in addition, at present, the BKF and SEN strains are separated by a natural barrier preventing gene flow, and thus evolve independently [10, 27, 43, 66, 67], with likely consequences for behavior [68] and physiology between the three strains that could explain the observed differences in their ability to survive at high temperatures. From an ecology or evolutionary point of view, distinguishing between strains on the basis of the survival at a given set of temperatures could present some limits and some other studies of thermal tolerance (e.g. critical thermal maximum or heat knockdown time) should be conducted to complement the survival assays presented here. Therefore, the absence of significant differences in survival between strains at a given temperature (e.g. at 26.4 °C) should be interpreted with caution, since a three-day difference in survival is considered substantial.

The difference in establishment time of the three strains suggests an adaptation to breeding conditions for BKF dating back more than four decades [45, 47], while SEN and SENbkf have been in culture for only five years (since 2010) [19]. This hypothesis is supported by data from the IPCL insectary, where the productivity of SEN flies at 25 ± 1 °C and 75 ± 5 % rH after four years of establishment reached the same level than that of BKF for fecundity but not for survival.

Genetically, the SENbkf strain contains 3 % of the nuclear genome of BKF and 97 % of the genome of SEN, whereas its mitochondrial DNA is 100 % BKF (maternal transmission). As for the relationship between the survival rate of females for the three strains and maximum temperature (see Fig. 4), the intermediate survival ability of SENbkf females at high temperatures might be related to the influence of mitochondrial DNA transmitted by the BKF strain. Further research is required to confirm the validity of this hypothesis.

In view of the performance of the BKF strain with respect to temperature variation, its use in the *G. p. gambiensis* eradication campaign in the Niayes area is justified, as the environmental conditions prevailing in the target area, i.e. 25–30 °C and 60–80 % rH [17] correspond to the optimal conditions for survival and competitiveness of this strain. This was confirmed in field pilot trials where released BKF sterile males showed good competitiveness in most ecosystems of the Niayes (unpublished data). In addition, only colonies of this strain were large enough to produce the weekly number of required sterile males for release in the Niayes.

At 25–26 °C and with a rH ranging from 40 to 76 %, we observed no effect of humidity on survival of the flies regardless of the strain, indicating that the different humidity conditions appeared to have no effect on the metabolism of the flies. This might be due to the rather small range of humidity conditions tested in our experiment. These results, however, are in agreement with the findings by other authors who observed that at 24 °C *G. f. fuscipes* lost ~ 0.47 mg fat in 24 h at rH levels of 19–88 % [63, 69] and Jackson [69] observed that at 25 °C the fat loss in 24 h for *G. palpalis*, *G. morsitans* and *G. swynnertoni* teneral males was comparable within a range of 19–88 % rH. A predictive study using a physiological and climate GIS database showed an effect of moisture on the physiology of *Glossina* pupae but not adults [70]. The lack of any effect of humidity on fly physiology was observed in other insects. Fasting bed-bugs, kept for various lengths of time at five different temperatures, ranging from 8–37 °C, and at different rH (i.e. 0, 30, 60 and 90 %), used the same amounts of food reserves at each humidity level for a given temperature, though more water evaporated from insects kept in dry air than in humid air [71].

Our results indicate that at 26 °C, the maximum temperature for the mass-rearing of BKF flies, the mean survival of this strain was 50 days [45–47]. By comparison, a mean survival of 50 days corresponded to a temperature of 25.6 and 24.9 °C for the SENbkf and SEN strains, respectively. Considering that 25 ± 1 °C is the optimal temperature for BKF mass-rearing [45–47], those of SENbkf and SEN might be slightly lower; however, a study of the fecundity at temperatures lower than 25 °C is necessary to determine the optimal rearing conditions for these strains. The optimal temperature for the mass-rearing of the three strains was relatively similar since the difference was less than ± 1 °C. Nevertheless, preliminary data (from the insectaries of origin) indicating that the mortality of SEN and SENbkf flies at 25 ± 1 °C and 75 ± 5 % rH was greater than that of BKF, support the hypothesis that the difference in establishment time of the three strains has resulted in differing levels of adaptation to breeding conditions. The optimal relative humidity for the BKF strain of 75 ± 5 % can also be considered valid for the two other strains, since a range from 40 to 76 % rH did not affect the survival and the fecundity of the three strains.

Above 30 °C, the survival of flies was too low to assess fecundity. Previous laboratory studies on *G. f. fuscipes* showed that a constant temperature of 30 °C caused sterility in females, with abnormal development of the ovaries and embryos failing to hatch from the eggs [72], similar to the the changes that occur when a tsetse female is deprived of *Wigglesworthia* [73]. In the same way, *G. pallidipes* pupae kept at 31 °C resulted in non-viable flies [74]. Below 30 °C, the only temperature where the fecundity was assessed was 25–26 °C; therefore, the effect of temperatures between 26 and 30 °C could not be evaluated and additional experiments are needed.

The rH (40-76 % at 25–26 °C) had no effect on the reproduction of the three *G. p. gambiensis* strains. The same result was obtained with *G. morsitans* [64] and *G. f. fuscipes* [63], where insemination rates and pupae production were affected by temperature but not by humidity.

Our data indicate that at 25–26 °C and 40-76 % rH female *G. p. gambiensis* produce their first larva at ~ 19 days old. These results do not differ significantly from those obtained in previous studies. Pollock [75] observed that the tsetse female (irrespective of the species) at 25 °C produces her first larval on day 18 to 20 post-emergence. According to Mellanby [72], the development of the first *G. f. fuscipes* larva takes a minimum of 18 to 19 days at 24 °C, made up of a minimum of eight days for oocyte maturation, three to four days for embryonic development and seven days for larval growth in the uterus, after which the mature larvae is deposited. But at 21–23 °C ovulation in *G. morsitans* and *G. swynnertoni* (morsitans group) was delayed to about the 12^th^ or 13^th^ day [64, 76].

At 25–26 °C and 40–76 % rH, a mean interlarval period of 8.5 days was observed for the BKF strain and ten days for the SEN and SENbkf strains. These results show that the interlarval period of BKF was shorter than observed for other species, but the observations for the SEN and SENbkf strains were in line with previous data obtained under stable insectary conditions, i.e. an interlarval period of ten days at 26 °C was obtained for *G. palpalis palpalis* [77], a mean of 9.9 days (range of nine to 11 days) at 24 °C for *G. f. fuscipes* [72], an interlarval period of 11 days at 24 °C for *G. morsitans* [64] and 13–14 days at 24–26 °C for *G. tachinoides* [49]. In view of the length of time that the BKF strain already spends in under insectary rearing, the resultant selection has promoted fecundity, as the BKF strain performed better than the two other strains.

Models developed using field data to predict the first larval and interlarval periods of *G. pallidipes* and *G. morsitans* [78, 79] indicated that the first larval period was slightly shorter for flies under field conditions as compared with laboratory flies, but there was no difference for the subsequent interval periods [39]. The estimated time to produce the first larva was between 14–17 days post-emergence depending on the temperature, i.e. at 25 °C the predicted time was 15.9 days [39, 80]. The difference between the first larval period under laboratory and field conditions might be due to the specific behavior of tsetse flies in the field, where they minimize the effects of extreme temperatures by using microenvironments, i.e. refuges when the temperatures are high, and resting in direct sunshine at low temperatures [79]. Previous findings have shown that in the field, tsetse appear to live at temperatures 2 to 6 °C lower than the room temperature (corresponding to the constant temperature of the laboratory) [81]. For this reason, caution must be taken in the interpretation of results predicted from field experiments.

## Conclusions

The survival and pupae production of *G. p. gambiensis* flies appeared to be governed mainly by temperature, and was unaffected by changing humidity within the explored range. The BKF strain survived at higher temperatures better than the SEN and SENbkf strains but the temperature limit of survival remained at about 32 °C for all strains.

## Abbreviations

AAT: African animal trypanosomoses
AICc: Corrected Akaike information criterion
AW-IPM: Area-wide integrated pest management
BKF: Burkina Faso
CIRAD: Centre de Coopération Internationale en Recherche Agronomique pour le Développement
CIRDES: Centre International de Recherche-Développement sur l’Elevage en zone Subhumide
CRTA: Centre de Recherche sur les Trypanosomiases Animales
FAO/IAEA: Food and Agriculture Organization/International Atomic Energy Agency
HAT: Human African trypanosomoses
IPCL: Insect pest control laboratory
PATTEC: Pan-African tsetse and trypanosomosis eradication campaign
PPIF: Pupae per initial female
rH: Relative humidity
SAS: Slovak Academy of Sciences
SD: Standard deviation
SEN: Senegal
SENbkf: Introgressed strain (SEN × BKF)
SIT: Sterile insect technique
